# Elevated serum autotaxin levels and multiple system atrophy-like presentation in a patient with *PLA2G6*-associated neurodegeneration

**DOI:** 10.1038/s10038-025-01342-0

**Published:** 2025-04-22

**Authors:** So Okubo, Takashi Matsukawa, Norifumi Kawamoto, Masahiko Tsujita, Kenta Orimo, Hiroya Naruse, Jun Mitsui, Masashi Hamada, Wataru Satake, Tatsushi Toda

**Affiliations:** 1 Department of Neurology, Graduate School of Medicine, The University of Tokyo, Tokyo, Japan; 2 Department of Precision Medicine Neurology, Graduate School of Medicine, The University of Tokyo, Tokyo, Japan

**Keywords:** Neurodegeneration, Neurodegeneration

## Abstract

*PLA2G6*-associated neurodegeneration (PLAN) encompasses a spectrum of phenotypes caused by biallelic pathogenic variants in *PLA2G6*. Initially linked to infantile and atypical neuroaxonal dystrophy, PLAN now includes adult-onset conditions such as dystonia-parkinsonism, ataxia, and spastic paraplegia. We report a female patient presenting young-onset parkinsonism with pyramidal tract signs, cerebellar atrophy, and autonomic dysfunction, mimicking multiple system atrophy (MSA). Neuroimaging showed decreased dopamine uptake and cerebellar hypoperfusion. Genetic analysis identified a homozygous pathogenic variant in *PLA2G6* (c.967G>A, p.Val323Met), confirming a diagnosis of PLAN. Interestingly, elevated serum autotaxin levels (4.67 ng/mL) without liver abnormalities. Bilateral brachymetatarsia was also observed, which may indicate an association with the *PLA2G6* variant. This case underscores the importance of considering PLAN in cases of young-onset parkinsonism with multisystem involvement. Genetic testing is crucial for accurate diagnosis and management of such cases. Elevated serum autotaxin levels may be associated with decreased phospholipase activity in PLAN and warrants further investigation.

## Introduction

*PLA2G6* encodes calcium-dependent phospholipase A_2_β, and biallelic pathogenic variants in this gene were initially associated with infantile neuroaxonal dystrophy and atypical neuroaxonal dystrophy, both of which manifest during infancy or early childhood [1, 2]. Subsequent studies have associated *PLA2G6* variants with various adult-onset phenotypes [3–6]. These phenotypes are collectively categorized as *PLA2G6*-associated neurodegeneration (PLAN).

Here, we present a case of PLAN presenting parkinsonism, pyramidal tract signs, autonomic dysfunction, and cerebellar atrophy, resembling multiple system atrophy (MSA). This patient showed elevated serum autotaxin levels, which may serve as a potential biomarker for PLAN.

## Case presentation

**Fig. 1:**
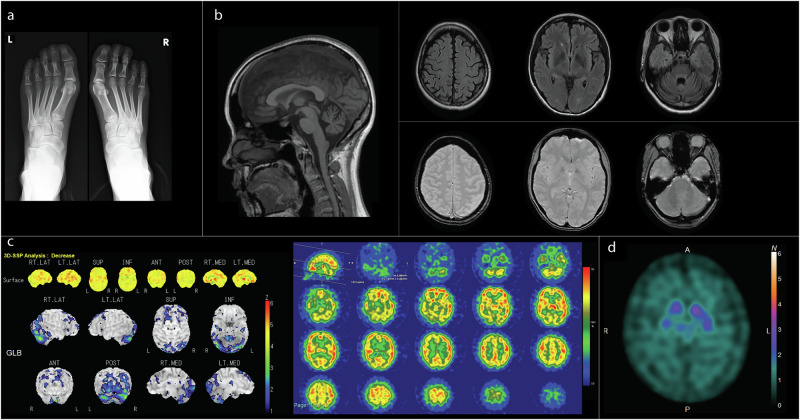
Imaging studies of the patient. **a** Plain radiographs of both feet. **b** Magnetic resonance imaging of the brain. Sagittal view of T1-weighted imaging (left) shows cerebellar atrophy. Fluid-attenuated inversion recovery (top right) shows no abnormal high-intensity lesions. T2*-weighted imaging (bottom right) shows no evidence of iron deposition. **c** Single-photon emission computed tomography with ^123^I-labeled iofetamine. Cerebellar blood flow is decreased. **d** Dopamine transporter scintigraphy. Decreased uptake, predominantly on the right side, was observed. The uptake was bean-shaped, not the dot-shaped uptake typically observed in Parkinson’s disease

A 29-year-old Hawaiian female patient was admitted to our institution for evaluation of gait difficulties, which had progressively worsened over a two-year period despite treatment with levodopa-carbidopa. Neurological examination showed parkinsonism characterized by soft and monotonous speech, left-sided dominant cogwheel rigidity, resting tremor, a positive Myerson sign, and a small-step gait. Additional findings included exaggerated jaw jerk, bilaterally exaggerated tendon reflexes, and positive Chaddock signs. Constipation and urinary incontinence were present but without orthostatic hypotension. Bilateral brachymetatarsia of the fourth toes was noted and confirmed on radiographs (Fig. 1a). Blood tests, performed prior to genetic analysis, revealed markedly elevated serum autotaxin levels (4.67 ng/mL, reference values 0.56–1.21 ng/mL) with no evidence of liver dysfunction. Autotaxin levels were not measured in the cerebrospinal fluid. Motor evoked potentials showed bilaterally prolonged central motor conduction time. Magnetic resonance imaging showed cerebellar atrophy without evidence of iron deposition (Fig. 1b). Single-photon emission computed tomography with ^123^I-labeled iofetamine showed decreased cerebellar blood flow (Fig. 1c). Dopamine transporter scintigraphy showed decreased uptake, predominantly on the right side (Fig. 1d). Cardiac ^123^I-metaiodobenzlguanidine (MIBG) scintigraphy did not show decreased uptake.

Although the patient’s family history was negative for consanguinity or neurological diseases, the young-onset of symptoms prompted consideration of a monogenic disease, particularly one with autosomal recessive inheritance. After obtaining written informed consent, the patient underwent genetic analysis.

**Fig. 2:**
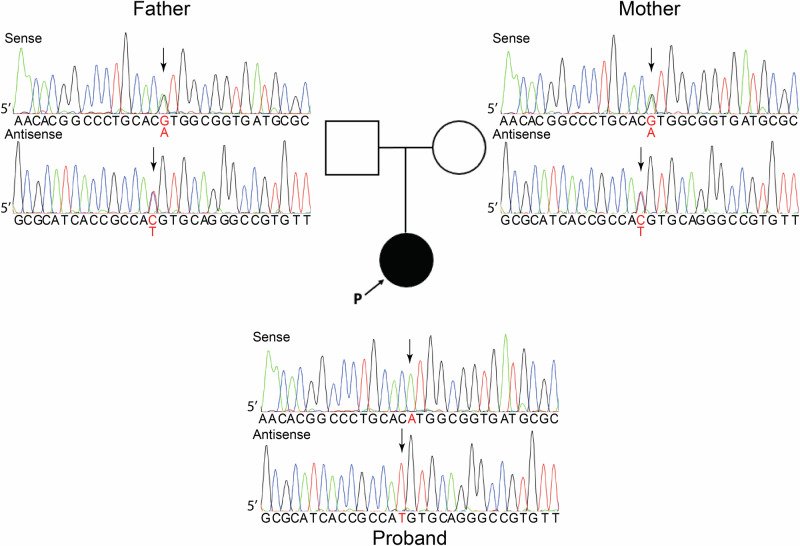
Electropherograms of the direct nucleotide sequencing analysis of the proband and both parents. The c.967G>A variant was homozygous in the proband, and heterozygous in both parents

Whole-exome sequencing was performed as detailed in the Supplementary methods. Rare variants in parkinsonism-related genes were screened, focusing on missense, nonsense, splice-site, and indel variants with a minor allele frequency of less than 0.01 in population databases, including the East-Asian gnomAD (https://gnomad.broadinstitute.org) and the ToMMo 60KJPNSNV/INDEL Allele Frequency Panel (https://jmorp.megabank.tohoku.ac.jp), both accessed in October 2024. This analysis identified a homozygous variant (c.967G>A (p.Val323Met)) in *PLA2G6* (NM_003560.4), which was previously reported as a pathogenic variant [7]. Direct nucleotide sequence analysis using an ABI 3130 genetic analyzer confirmed this homozygous variant in the patient with the specific primers described in Supplementary Table 1. Subsequent analysis confirmed that both parents were heterozygous carriers (Fig. 2). From these findings, a diagnosis of PLAN was established.

## Discussion

The adult-onset form of PLAN was originally reported as a dystonia-parkinsonism syndrome [3]. In subsequent reports, additional phenotypes were identified, such as ataxia and complicated forms of spastic paraplegia, indicating the broad spectrum of presentations associated with *PLA2G6* variants [4, 5]. Moreover, overlapping phenotypes have been documented in certain cases [6]. According to a recent review, the prevalences of pyramidal tract signs, cerebellar atrophy, and autonomic dysfunction were 77.2%, 46.5%, and 71.4%, respectively [8]. This shows that multiple-system involvement of the nervous system, as observed in this case, is relatively common in PLAN. Available information on neuropathology included Lewy body accumulation in the substantia nigra and cerebellar cortical atrophy [8], supporting the multiple-system involvement. The mechanism of autonomic dysfunction remains unclear based on pathological studies. Given that the majority of cases, including the present one, do not exhibit abnormal MIBG scan findings, mechanisms other than postganglionic sympathetic denervation may be involved. The current Movement Disorder Society criteria for the diagnosis of MSA require onset after the age of 30. Thus, PLAN patients, whose symptoms typically manifest before this age, would rarely fulfill these criteria. Nevertheless, young-onset parkinsonism mimicking MSA should warrant investigation of PLAN through broad genetic testing such as whole-genome sequencing or whole-exome sequencing.

A notable feature of this patient was the elevated levels of serum autotaxin. Autotaxin is a soluble extracellular enzyme that plays a crucial role in the lysophosphatidic acid (LPA) signaling pathway by hydrolyzing extracellular lysophosphilipids into the lipid mediator LPA [9]. Autotaxin is expressed in various tissue including the brain and spinal cord, small intestine, spleen, lung, kidney, uterus, and ovaries. The highest expression is observed in the spinal cord, according to GTEx portal (https://gtexportal.org/, last accessed March 2025). The LPA pathway has a wide variety of functions ranging from cell proliferation, survival, and wound healing. In the nervous system, its physiological functions include brain development, neuronal differentiation, oligodendrocyte development, and microglial activation [9]. Serum autotaxin levels are widely utilized as a biomarker to predict inflammation activity and fibrosis in various liver diseases [10].

**Fig. 3:**
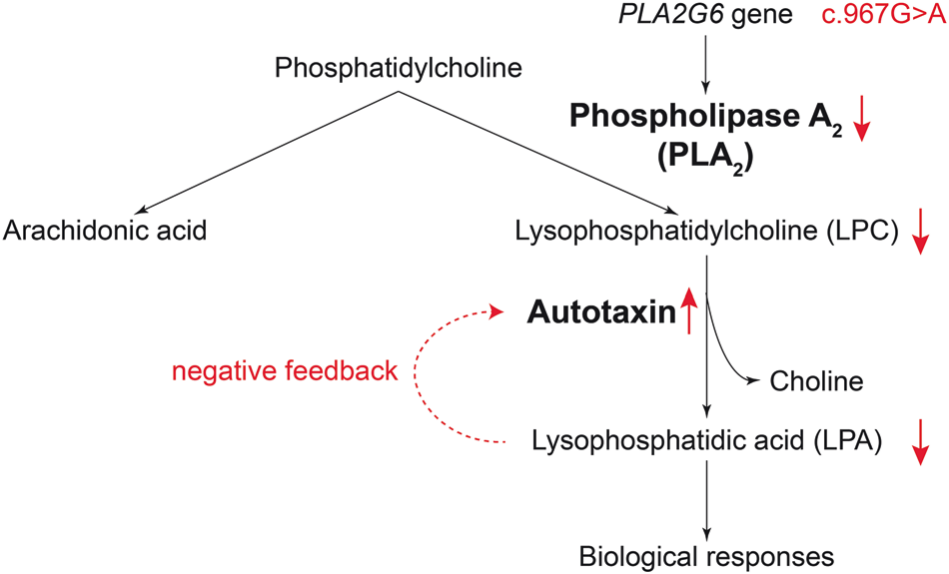
Schematic overview of the lysophosphatidic acid (LPA) signaling pathway. Changes associated with decreased PLA2G6 activity in our proposed hypothesis are shown in red. LPA lysophosphatidic acid, LPC lysophosphatidylcholine, PLA_2_ phospholipase A_2_

Based on the function of PLA2G6 (phospholipase A2 group VI), we hypothesized that elevated serum autotaxin levels would be observed in PLAN, prompting us to measure serum autotaxin while awaiting genetic analysis. Accordingly, serum autotaxin levels were markedly elevated without evidence of liver dysfunction in this patient. PLA2G6 contains seven ankyrin-like repeats, spanning amino acid positions 151 to 382, which facilitate the oligomerization of the enzyme, a crucial step for full enzymatic activity [11]. The p.Val323Met variant identified in this patient resides within the sixth ankyrin-like repeat. Previous studies have shown that other variants located in the same region, such as p.Asp331Tyr [12] and p.Ala341Thr [13] variants, cause decreased enzymatic activity of PLA2G6. The c.967G>A (p.Val323Met) variant has a Phred score of 24.4 according to Combined Annotation Dependent Depletion version 1.7 [14], supporting its potentially deleterious impact. These findings suggest that the c.967G>A variant leads to reduced PLA2G6 activity. Given the role of PLA2G6 in the metabolic pathway of the LPA axis [9], the observed elevation in serum autotaxin levels could represent a compensatory mechanism in response to reduced upstream phospholipase activity (Fig. 3). Thus, elevated serum autotaxin levels could serve as a diagnostic biomarker in PLAN patients harboring *PLA2G6* variants that impair enzymatic function. Due to technical limitations, we were not able to quantify LPA and lysophosphatidylcholine (LPC). Further investigations, incorporating direct measurements of LPA and LPC, would be valuable for elucidating the association between PLAN and autotaxin.

Finally, this case demonstrated concurrent bilateral brachymetatarsia of the fourth toes. Brachymetatarsia can occur secondary to conditions such as sickle cell disease; however, the majority of cases are idiopathic, with an estimated prevalence of 0.02–0.05%, predominantly affecting females [15]. To our knowledge, there have been no previous reports describing brachymetatarsia in PLAN patients. Although this finding may be coincidental, one possibility is that brachymetatarsia represents a previously unrecognized manifestation of PLAN. Alternatively, idiopathic bilateral brachymetarsia may have occurred independently and coincided with PLAN owing to a homozygous biallelic variant in strong linkage disequilibrium with the identified variant of *PLA2G6*. Whole-exome sequence data were analyzed to search for such variants; however, numerous nonsynonymous homozygous variants were identified near the *PLA2G6* locus, making it challenging to draw definitive conclusions. Further observation of similar cases may provide insights into the potential genetic basis of idiopathic brachymetatarsia and its possible association with PLAN.

## Conclusion

Young-onset parkinsonism accompanied by autonomic dysfunction, cerebellar atrophy, and pyramidal dysfunction should prompt an investigation of PLAN. Due to the wide variety of differential diagnosis in such cases, broad genetic testing including whole-genome sequencing or whole-exome sequencing is essential for achieving accurate diagnosis and guiding appropriate management. Elevated levels of serum autotaxin may reflect decreased PLA2G6 activity in PLAN, highlighting their potential as a biomarker that warrants further investigation.

## Supplementary information

Supplementary methods

Supplementary Table
