# Supplementary material for: Elevated serum autotaxin levels and multiple system atrophy-like presentation in a patient with PLA2G6-associated neurodegeneration
Source: J Hum Genet. 2025 Apr 22;70(7):381–4. doi: 10.1038/s10038-025-01342-0 (PMC12137112; doi:10.1038/s10038-025-01342-0)
Supplement: Supplementary file 1 — Supplementary methods [file 10038_2025_1342_MOESM1_ESM.docx]

**Supplementary methods**

**Whole exome sequence**

Genomic DNA samples were extracted from peripheral blood mononuclear cells using standard procedures. The extracted genomic DNAs were subjected to enrichment of exonic sequences using the Agilent SureSelect Human All Exon V7 (Agilent Technologies, Santa Clara, CA, USA). Massively parallel sequencing was accomplished using NovaSeqX Plus (Illumina, 150 bp, paired end reads). After checking the sequencing quality with FastQC, the analysis was conducted according to the GATK best practices pipeline^1^. Burrows–Wheeler Alignment Tool^2^ was used with default parameter settings for the alignment of raw reads. Picard (<http://broadinstitute.github.io/picard>) was used to mark duplicates. GATK tools (<https://gatk.broadinstitute.org/>) were used for recalibration, the detection of single-nucleotide variants and short insertion/deletion variants (indels), and variant filtration. Variants were annotated with RefSeq (<https://www.ncbi.nlm.nih.gov/refseq/>), 1000 Genomes Project database (<https://www.internationalgenome.org>), and dbSNP 156 (<https://www.ncbi.nlm.nih.gov/snp/>).

**Parkinsonism-related genes**

As the disease-causative genes of parkinsonism, we searched for rare variants by whole-exome sequencing in *SNCA, UCHL1, LRRK2, GIGYF2, HTRA2, VPS35, EIF4G1, TMEM230, CHCHD2*, and *RIC3* with autosomal dominant inheritance genes, and *PRKN, PINK1, PARK7, ATP13A2, PLA2G6, FBXO7, DNAJC6, SYNJ1,* and *VPS13C* with autosomal recessive inheritance. Direct nucleotide sequence analysis confirmed that this patient did not harbor variants in *GBA1* associated with Parkinson’s disease.

**Supplemental references**

1. Van der Auwera GA, O'Connor BD. (2020). Genomics in the Cloud: Using Docker, GATK, and WDL in Terra (1st Edition). O'Reilly Media.

2. Li H, Durbin R. Fast and accurate short read alignment with Burrows–Wheeler transform. Bioinformatics. 2009;25:1754–1760.
