# Supplementary material for: Elevated serum autotaxin levels and multiple system atrophy-like presentation in a patient with PLA2G6-associated neurodegeneration
Source: J Hum Genet. 2025 Apr 22;70(7):381–4. doi: 10.1038/s10038-025-01342-0 (PMC12137112; doi:10.1038/s10038-025-01342-0)
Supplement: Supplementary file 2 — Supplementary Table [file 10038_2025_1342_MOESM2_ESM.docx]

**Supplementary Table 1.** Primers for direct nucleotide sequence analysis.

| **Primer name** | | **Sequence** | **Primer length** |
| --- | --- | --- | --- |
|  | PLA2G6_ex7_F | 5'-TGTGCTAACTCAGCCTGACC | 20 |
|  | PLA2G6_ex7_R | 5'-TCCTGGGCTCACCGACAT | 18 |
